# Impact of Semi-Annual Albendazole on Lymphatic Filariasis and Soil-Transmitted Helminth Infection: Parasitological Assessment after 14 Rounds of Community Treatment

**DOI:** 10.4269/ajtmh.21-0731

**Published:** 2021-12-20

**Authors:** Sébastien D. S. Pion, Cédric B. Chesnais, Gary J. Weil, Frédéric Louya, Michel Boussinesq, François Missamou

**Affiliations:** ^1^ Institut de Recherche pour le Développement, UMI233/INSERM U1175/Université de Montpellier, Montpellier, France;; ^2^ Infectious Diseases Division, Washington University School of Medicine, St. Louis, Missouri;; ^3^ Programme National de Lutte contre l’Onchocercose, Direction de l’Epidémiologie et de la Lutte contre la Maladie, Ministère de la Santé et de la Population, Brazzaville, Republic of Congo

## Abstract

Between October 2012 and October 2015, we conducted a community trial to assess the impact of semi-annual (twice yearly) community treatment with albendazole on lymphatic filariasis in Seke Pembe, a village in the Republic of the Congo. Semi-annual community treatment with albendazole has been continued in the community since October 2015. We conducted an additional parasitological assessment survey in October 2019, 6 months after the 14th round of semi-annual treatment. Between October 2012 and October 2015, *Wuchereria bancrofti* antigenemia and microfilaremia rates in the community had decreased from 17.3% to 4.7% and from 5.3% to 0.3%, respectively. In October 2019, the antigenemia rate had decreased further to 2.8% (19 of 687). No microfilariae were found in night blood smears from persons with circulating filarial antigenemia (0 of 16), suggesting that *W. bancrofti* transmission has been interrupted in Seke Pembe. Semi-annual albendazole treatments also reduced significantly infection rates with soil-transmitted helminths.

Loiasis, the filarial infection caused by the worm *Loa loa* is endemic in central Africa. In addition to the fact that *L. loa* causes significant morbidity, it also represents a major impediment to the implementation of onchocerciasis and lymphatic filariasis (LF) elimination programs. Those elimination programs rely on repeated mass drug administration (MDA) with ivermectin alone for onchocerciasis and ivermectin plus albendazole for LF. However, individuals infected heavily with *L. loa* are at risk of developing serious adverse events when treated with ivermectin.^1^ Since 1990, more than 500 cases of characteristic encephalopathy after ivermectin treatment, including approximately 60 fatal cases, have occurred during MDA and have been reported to the Mectizan Donation Program.^2^ This led the WHO to define a specific strategy to implement LF elimination programs safely in areas where loiasis is endemic and onchocerciasis is hypoendemic. In 2012, the WHO proposed a provisional strategy for the interruption of transmission of LF comprised of MDA with albendazole alone (400 mg every 6 months) together with integrated vector management to control mosquitoes.^3^

Before the WHO provisional policy was announced, we had started independently, in October 2012, a community trial of semi-annual (i.e., twice yearly) mass administration of albendazole in Seke Pembe, a village in Republic of the Congo.^4^ Seke Pembe has a total population of about 900 individuals. All consenting inhabitants 2 years or older were offered albendazole (400 mg) every 6 months. Infection with *Wuchereria bancrofti* was diagnosed with the BinaxNOW Filariasis card immunochromatographic test (Alere, Scarborough, ME) that detects circulating filarial antigenemia (CFA). People with CFA were tested for microfilaremia by night blood smears. Individuals were also tested for soil-transmitted helminth (STH) infections (i.e., hookworm, *Ascaris lumbricoides*, and *Trichuris trichiura*) with the Kato-Katz method.

The trial was planned initially for 3 years, and parasitological assessments were scheduled to take place once per year, just before albendazole MDA. Therefore, assessment surveys were done in October 2013, October 2014, and October 2015. The main outcome measure was change in infection rates from baseline to year 3. Between 2012 and 2015, *W. bancrofti* antigenemia and microfilaremia rates in the community decreased significantly, from 17.3% to 4.7% and from 5.3% to 0.3%, respectively. Semi-annual MDA with albendazole also had a dramatic impact on STH infections. No hookworm infections were detected 6 months after the second round of MDA in October 2013. Between 2012 and 2015, the prevalence of *A. lumbricoides* infection decreased by 77.2% (from 56.5% to 12.9%) and that of *T. trichiura* by 24.4% (from 78.6% to 59.4%).

The Congolese National Neglected Tropical Diseases Control Program (NTD-CP), which was involved in the community trial since the outset, has been in charge of continuing semi-annual albendazole MDA in the trial community since October 2015. Between October 2012 and October 2015, the therapeutic coverage, defined as the population treated among those 2 years or older exceeded 80%.^4^ The therapeutic coverage remained at more than 75% for each round of MDA provided by the NTD-CP after 2015. Thanks to a funding extension, we were able to conduct an additional parasitological assessment survey in October 2019, 6 months after the 14th round of semi-annual community treatment.

We tested 688 persons (303 males and 385 females) 5 years or older (median age, 21 years; interquartile range, 11–42 years) out of a total population of 865. CFA was detected with the Filariasis Test Strip (FTS) (Alere, Scarborough, ME), which replaced the immunochromatographic test and is more sensitive than the latter.^5^ One person had an invalid result because of failure of blood migration. Therefore, FTS results were available for 687 individuals. Nineteen individuals (2.8%) had a positive FTS results; this included 17 who had positive CFA tests in prior rounds, and two individuals who were tested for the first time. The youngest person with a positive CFA test in 2019 was 21 years old (Figure 1). Thus, we detected no new infections in 2019 among persons who had been tested in the study that ended in 2015. This result suggests that *W. bancrofti* transmission has been interrupted in Seke Pembe and that improvements observed through 2015 were sustained by MDA provided by the Congolese National NTD-CP after that time.

**Figure 1.**
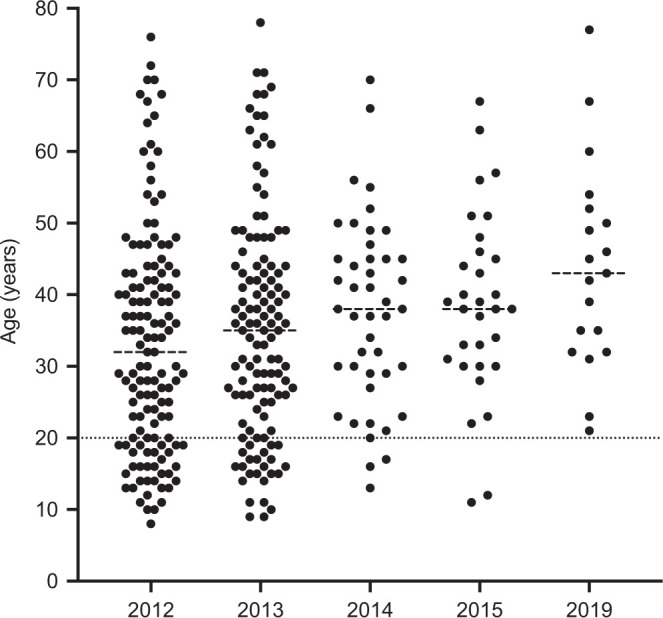
Age distribution of circulating filarial antigenemia-positive persons during the 7-year follow-up.

Night blood smears were collected from 16 of 19 persons with CFA (Figure 2). One individual was missing and two refused the night blood sampling. No microfilariae were found in night blood smears from 16 persons with CFA (two 70-µL smears per person). This supports the hypothesis that persons with CFA are unlikely to support LF transmission. Bed net use may have also contributed to LF elimination in Seke Pembe. A large majority (79%) of FTS-positive subjects stated they regularly slept under a bed net. However, community health workers reported that bed nets were last distributed in the area in 2009 and 2010 through the National Malaria Control Program. Thus, bed nets are likely to have been only partially effective in recent years. In addition, the finding that hotspots of *W. bancrofti* transmission for Seke Pembe residents were likely outside of the village^6^ would also tend to reduce the impact of bed net use. Most importantly, recently published studies of the efficacy of repeated doses of albendazole for clearing CFA and microfilariae provide very strong evidence that MDA with albendazole was primarily responsible for improved LF parameters in Seke Pembe.^7^^,^^8^

In 2019, 383 participants (162 males; 221 females; median age, 23 years; interquartile range, 11–47 years) provided a stool sample for STH infection assessment. The prevalence of *A. lumbricoides* infections was 13.6%, a value similar to that observed in 2015 (12.9%, Figure 3A). The arithmetic mean number of *A. lumbricoides* eggs per gram of feces (epg) decreased from 724 in 2015 to 589 epg between 2015 and 2019. *Trichuris trichiura* prevalence was 42.9% in 2019, which is moderately lower than the 59.4% measured in 2015 (Figure 3B). The arithmetic mean number of *T. trichiura* epg decreased from 356 epg in 2015 to 276 epg in 2019. The persistence of *T. trichiura* in the community was expected because this parasite is poorly sensitive to albendazole. Hookworm prevalence was approximately 5% when our study started in 2012. Hookworm infections were not detected after 2013 in our study, and no hookworm infections were detected in 2019 (Figure 3C). These results suggest that improvements in STH infection prevalence and intensity observed between 2012 and 2015 have been sustained since that time by the government’s MDA program.

**Figure 2.**
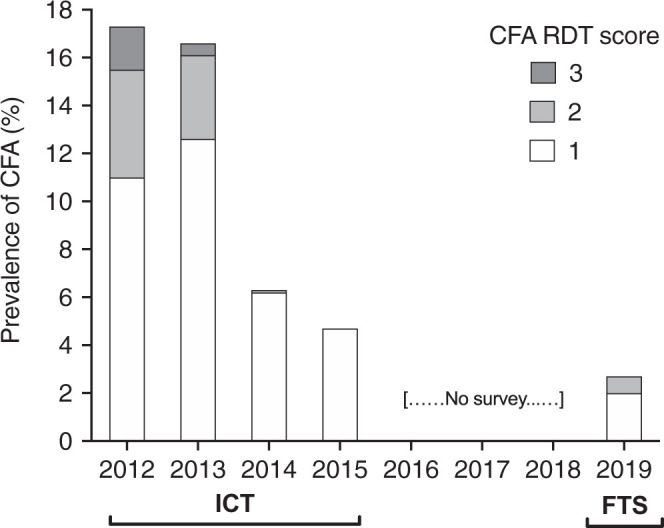
*Wuchereria bancrofti* circulating filarial antigenemia (CFA) score distribution during the 7-year follow-up. Rapid diagnostic test (RDT) results were scored semi-quantitatively as follows: negative tests with no visible test (T) line were assigned a score of 0 point, tests with a clearly visible T line that was weaker than the control (C) line were assigned a score of 1 point, tests with a T line approximately as dark as the control line were scored as 2 points, and cards with a T line darker than the C line were scored as 3 points. FTS = Filariasis Test Strip; ICT = immunochromatographic test.

**Figure 3.**
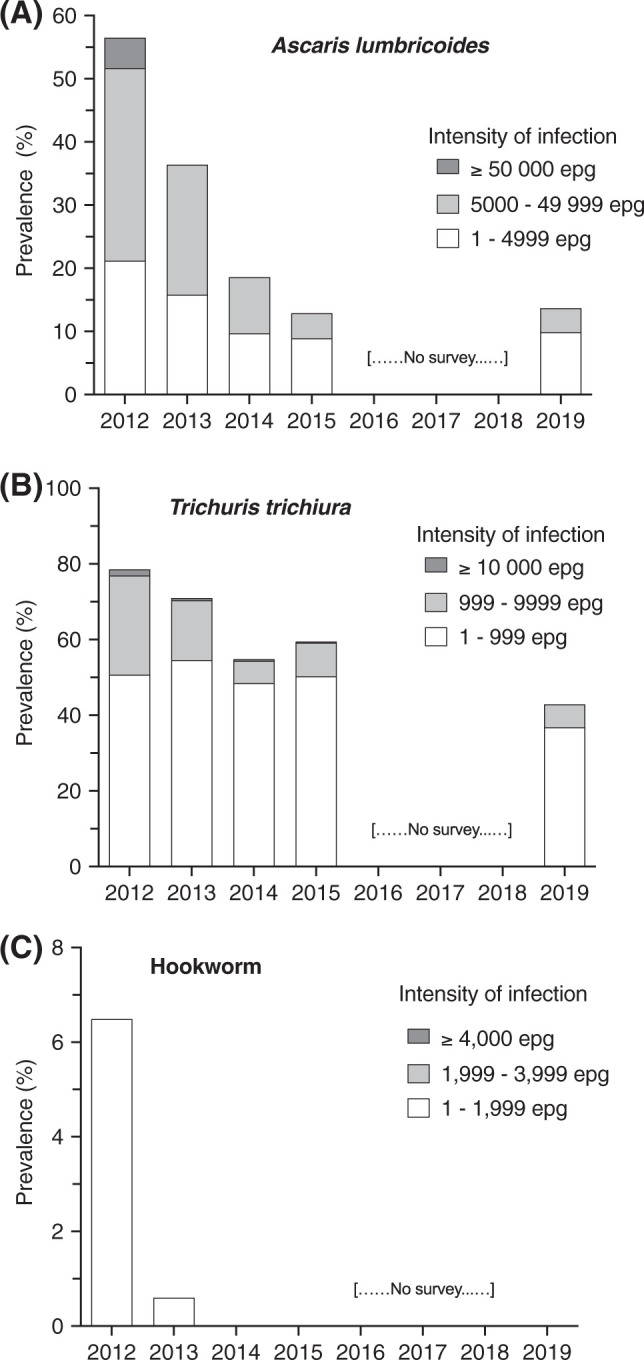
*Ascaris lumbricoides*, *Trichuris trichiura*, and hookworm infections during the 7-year period. Infection intensities were classified as heavy, moderate, or light according to WHO guidelines. epg = eggs per gram of feces.

This parasitological assessment, conducted after 14 semi-annual rounds of community treatment with albendazole and at high therapeutic coverage rates shows that the WHO provisional strategy can interrupt LF transmission in areas with moderate endemicity. Although MDA was also effective for controlling STH, complementary actions are needed to achieve STH elimination.
